# The Value of Information Searching against Fake News

**DOI:** 10.3390/e22121368

**Published:** 2020-12-03

**Authors:** José Martins, Alberto Pinto

**Affiliations:** 1 LIAAD-INESC TEC and School of Technology and Management, Polytechnic of Leiria, Campus 2, Morro do Lena-Alto do Vieiro, 2411-901 Leiria, Portugal; 2 LIAAD-INESC TEC and Faculty of Sciences, University of Porto, R Campo Alegre, 4169-007 Porto, Portugal; aapinto@fc.up.pt

**Keywords:** fake news, rumor spreading, Nash equilibrium, evolutionarily stable strategies, evolutionary information search dynamics

## Abstract

Inspired by the Daley-Kendall and Goffman-Newill models, we propose an Ignorant-Believer-Unbeliever rumor (or fake news) spreading model with the following characteristics: (i) a network contact between individuals that determines the spread of rumors; (ii) the value (cost versus benefit) for individuals who search for truthful information (learning); (iii) an impact measure that assesses the risk of believing the rumor; (iv) an individual search strategy based on the probability that an individual searches for truthful information; (v) the population search strategy based on the proportion of individuals of the population who decide to search for truthful information; (vi) a payoff for the individuals that depends on the parameters of the model and the strategies of the individuals. Furthermore, we introduce evolutionary information search dynamics and study the dynamics of population search strategies. For each value of searching for information, we compute evolutionarily stable information (ESI) search strategies (occurring in non-cooperative environments), which are the attractors of the information search dynamics, and the optimal information (OI) search strategy (occurring in (eventually forced) cooperative environments) that maximizes the expected information payoff for the population. For rumors that are advantageous or harmful to the population (positive or negative impact), we show the existence of distinct scenarios that depend on the value of searching for truthful information. We fully discuss which evolutionarily stable information (ESI) search strategies and which optimal information (OI) search strategies eradicate (or not) the rumor and the corresponding expected payoffs. As a corollary of our results, a recommendation for legislators and policymakers who aim to eradicate harmful rumors is to make the search for truthful information free or rewarding.

## 1. Introduction

The theory of rumor (or fake news) spreading proposed by Daley and Kendall [1,2] became known as the DK model, in which a population is divided into three different groups: ignorants—people who are ignorant concerning the rumor; spreaders—people who actively spread the rumor; and stiflers—people who have heard the rumor but are no longer interested in spreading it. Goffman and Newill [3] also published a paper in 1964 that generalized epidemic theory and provided a clear analogy between the spreading of infectious disease and the transmission of ideas. In the subsequent years, several authors developed the theory of rumor spreading, proposing new models using complex networks [4], transitions capable of describing different issues in the transmission process [5], and Lévy noise [6]. In this paper, we develop a game-theoretical approach for a network-extended version of the rumor spreading models proposed in [1,3], using the ideas developed by Bauch and Earn [7] for the well-known Susceptible-Infected-Recovered (SIR) epidemiological model. Furthermore, the topic of fake news is quantified in terms of the value of searching for truthful information (learning), the impact of believing the fake rumor, and the individual’s payoff, which is of paramount importance in academia.

Rumors and fake news can be considered a form of cheating. Individuals might be pushed toward risk-seeking or loss aversion on the basis of their feelings (see [8]). Political news can have a strong effect on stock prices (see [9]). In terms of the outbreak of COVID-19, information on social media can lead to numerous negative behaviors that can reduce vaccination coverage and the use of COVID alert applications (see [10]). On the other hand, rumors and fake news do not necessarily have negative impacts. An extreme example occurred during the Cold War: the propaganda machines from the American and Soviet sides spread numerous rumors about (i) the intentions of their rivals and (ii) the achievements of their countries in several areas (e.g., science, business, and industry). These rumors served the purpose of contributing to improvements in the well-being of both populations. If we see a rumor as an exaggerated piece of information with an essence of truth, then it can have a positive impact on the population. Another example occurred during World War II, when many rumors were spread concerning Nazi Germany. Although some news was fake, it served the purpose of boosting the morale of the Allied population and the troops. Hence, fake news can have either a negative or a positive impact on an individual’s behavior.

In the Ignorant-Believer-Unbeliever (IBU) rumor (or fake news) spreading dynamical model, individuals are spatially distributed in a network and can be either ignorants, believers, or unbelievers regarding a certain rumor. When a rumor appears in a population, individuals will act differently depending on their beliefs about the rumor. If an individual believes the rumor, then he/she will spread the rumor to his/her neighbors. On the other hand, individuals who do not believe the rumor will not act as active spreaders. This spreading dynamical model is fully inspired by the SIR epidemiological model. The impact measure *y* of the rumor evaluates the gains and losses resulting from individuals’ decisions, provoked by their beliefs in the rumor. The value *v* of searching for truthful information (learning), instead of just believing the rumor, has natural benefits and costs to the individual. Each ignorant individual has his/her own information search strategy *S* based on his/her probability of searching for truthful information per unit of time. The population’s information search strategy *s* is the proportion of ignorant individuals who will choose to search for truthful information per unit of time. For instance, if all ignorant individuals follow the same strategy *S* (homogeneous strategy), then s=S. For an ignorant individual, we introduce the expected information search payoff, which depends on (i) his/her information search strategy *S*; (ii) the population information search strategy *s*; (iii) the value *v* of searching for truthful information; (iv) the impact measure *y* of the rumor; and (v) the spread dynamics of the rumor.

A population information search strategy *S* is a Nash equilibrium if not a single individual has an incentive to change his/her information search strategy to any other strategy $S^{\prime }\neq S$ (see [11]). A population information search strategy *S* is an evolutionarily stable information search strategy if any small group of individuals that tries to adopt a different strategy $S^{\prime }$ obtains a lower payoff than those adopting the original strategy *S* (see [11]). Evolutionarily stable information search strategies are Nash strategies that are practiced by individuals in non-cooperative environments. A population information search strategy *S* is an optimal information search strategy if it maximizes the payoffs of individuals. Optimal information search strategies are practiced by individuals in (eventually forced) cooperative environments. Here, we fully characterize the triples (v,y,S), where *S* is (i) a Nash strategy, (ii) an evolutionarily stable information search strategy, or (iii) an optimal information search strategy; *v* is the value of searching for information; and *y* is the impact measure of believing a false rumor (fake news). Finally, we introduce evolutionary information searching dynamics following the replicator dynamics theory [11,12,13], where the search strategies evolve over time to increase the payoffs of individuals. Evolutionarily stable information search strategies are the attractors of the dynamics; i.e., over time, the population search strategy tends toward the evolutionarily stable information search strategy (and not necessarily to the optimal information search strategies).

For rumors that are advantageous to the population (positive impact y>0), three distinct scenarios occur, depending on the value of searching for truthful information: (i) for high positive values of searching, both evolutionarily stable information (ESI) and optimal information (OI) search strategies coincide, and all individuals search for truthful information, eradicating the rumor; (ii) (bi-stability) for small positive values of searching, there are two ESI search strategies: either all individuals search for truthful information (eradicating the rumor in non-cooperative environments), or no one searches for truthful information (persistence of the rumor in non-cooperative environments), and the OI search strategy jumps (at the right-boundary of this bi-stability region) from no one searching (persistence of the rumor in cooperative environments) to all individuals searching (eradicating the rumor in cooperative environments); (iii) for negative values of searching, we show that both ESI and OI search strategies coincide, and no individuals search for truthful information, and thus, the rumor persists.

For rumors that are harmful to the population (negative impact y<0), we show the existence of three distinct scenarios that occur, depending on the value of searching for truthful information: (i) for positive values of searching, both ESI and OI search strategies coincide, and all individuals search for truthful information, eradicating the rumor; (ii) for small negative values of searching, the OI search strategy coincides with the critical probability that is necessary to eradicate the rumor, and thus, the rumor is eradicated in cooperative environments, but the ESI search strategy is less successful than the OI search strategy, so, unfortunately, the rumor persists in non-cooperative environments; (iii) for highly negative values of searching, both ESI and OI search strategies coincide, and no individuals search for truthful information, and thus, the rumor persists. Hence, a recommendation for legislators and policymakers who aim to eradicate harmful rumors is to make the search for truthful information free or rewarding, i.e., information search value v≥0. For instance, truthful public social media campaigns can help by making the information easily available.

This paper is organized as follows. In Section 2, we introduce the IBU rumor spreading model for networks. In Section 3, we introduce a utility for individuals that depends on the value of information and the impact of believing the rumor. Nash and evolutionarily stable information search strategies are completely characterized. In Section 4, optimal information search strategies are deduced for different values of information. In Section 5, we introduce evolutionary information search dynamics and study its attractors. Section 6 provides the conclusions of the paper and directions for future research work.

## 2. The IBU Spreading Model on Regular Networks 

Inspired by the work in [1,3], we propose the Ignorant-Believer-Unbeliever (IBU) dynamic model for rumor spreading based on the classical Susceptible-Infected-Recovered (SIR) epidemic model (see also [14,15]). Individuals can be either *Ignorants*, *Believers*, or *Unbelievers* of a certain rumor. The IBU model is directly analogous to the SIR model:

*S—Susceptibles* correspond to *I—Ignorants*,

*I—Infected* individuals correspond to *B—Believers* of the rumor,

*R—Recovered* individuals correspond to *U—Unbelievers* of the rumor.

**Figure 1:**
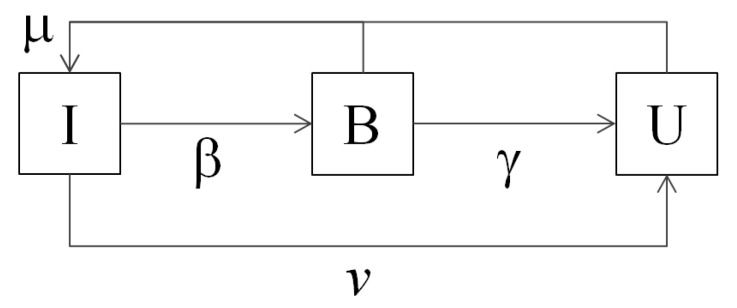
The compartmental Ignorant-Believer-Unbeliever (IBU) rumor spreading model.

Individuals who believe the rumor are the active spreaders: i.e., they are the individuals who transmit the rumor to ignorant individuals. Once a believer stops believing the rumor and becomes an unbeliever, he/she will stop transmitting the rumor. Hence, unbelievers are not active spreaders. As in epidemiology [10], a transition corresponding to vaccination is introduced in the model. This transition is due to information search activities that can be voluntarily adopted by an ignorant individual. State transitions in the IBU model are illustrated in Figure 1 and defined by the following reaction scheme:

$$\begin{array}{ccc} I_{i}+B_{j} & \overset{\beta }{⟶} & B_{i}+B_{j}\\ B_{i} & \overset{\gamma }{⟶} & U_{i}\\ I_{i} & \overset{\nu }{⟶} & U_{i}\\ I_{i},B_{i},U_{i} & \overset{\mu }{⟶} & I_{i}. \end{array}$$

The individual state variables $I_{i}$, $B_{i}$, and $U_{i}$∈{0,1} identify the state of individual *i*, restricted to the condition that the individual belongs to one of the three classes. Hence,

$$I_{i}+B_{i}+U_{i}=1.$$

The parameters of the model have the following interpretation: β is the rate at which one believer individual spreads the rumor; μ is the mean birth and death rates, and thus, 1/μ is the mean life expectancy at birth; γ is the rate at which a believer stops believing the rumor and stops spreading it, and thus, 1/(γ+μ) is the mean believing/spreading period; ν is the information search rate, i.e., the rate at which an ignorant individual searches for real information and become an unbeliever.

Let us assume that a population is fixed in size with *N* individuals; hence,

$$\sum_{i=1}^{N}\left(I_{i}+B_{i}+U_{i}\right)=N.$$

To describe the neighbor structure of individuals in the population, we consider the N×N adjacency matrix *J* with elements $J_{i,j}\in \left\{0,1\right\}$ such that: if individual *i* is a neighbor of *j*, then $J_{i,j}=1$, and if individual *i* is not a neighbor of *j*, then $J_{i,j}=0$. The matrix *J* is symmetric with zero elements in the diagonal. Let $\left\{I_{1},B_{1},U_{1},...,I_{i},B_{i},U_{i},...,U_{N}\right\}$ denote a certain state of the population, and let $p(I_{1},B_{1},U_{1},...,I_{i},B_{i},U_{i},...,U_{N},t)$ be the probability of that state occurring at time *t*. The time evolution of $p(I_{1},B_{1},U_{1},...,I_{i},B_{i},U_{i},...,U_{N},t)$ is described by a master equation [16] given by an ordinary differential equation (ODE) system that models the probabilistic combination of states and the switching between those states depending on the transition rates of the mathematical model and the spatial structure of the population. Following Glauber’s Ising spin dynamics [17] or Stollenwerk et al.’s reinfection SIRI model [18,19], the master equation for the IBU spreading model is given by

(1) $$\begin{array}{ccc} \frac{d}{dt} & p & \left(I_{1},B_{1},U_{1},...,I_{i},B_{i},U_{i},...,U_{N},t\right)\\ & = & \sum_{i=1}^{N}\beta \left(\sum_{j=1}^{N}\;J_{ij}B_{j}\right)\;\left(1-I_{i}\right)\;p\left(I_{1},B_{1},U_{1},...,1-I_{i},1-B_{i},U_{i}...,U_{N},t\right)\\ & & +\sum_{i=1}^{N}\gamma \;\left(1-B_{i}\right)\;p\left(I_{1},B_{1},U_{1},...,I_{i},1-B_{i},1-U_{i}...,U_{N},t\right)\\ & & +\sum_{i=1}^{N}\nu \;\left(1-I_{i}\right)\;p\left(I_{1},B_{1},U_{1},...,1-I_{i},B_{i},1-U_{i}...,U_{N},t\right)\\ & & +\sum_{i=1}^{N}\mu \;[\left(1-I_{i}\right)\;p\left(I_{1},B_{1},U_{1},...,1-I_{i},B_{i},U_{i}...,U_{N},t\right)\\ & & \;+\;\left(1-B_{i}\right)\;p\left(I_{1},B_{1},U_{1},...,1-I_{i},1-B_{i},U_{i}...,U_{N},t\right)\\ & & \;+\;\left(1-U_{i}\right)\;p\left(I_{1},B_{1},U_{1},...,1-I_{i},B_{i},1-U_{i}...,U_{N},t\right)]\\ & & -\sum_{i=1}^{N}\left[\beta \left(\sum_{j=1}^{N}\;J_{ij}B_{j}\right)I_{i}+\gamma \;B_{i}+\nu I_{i}+\mu \left(I_{i}+B_{i}+U_{i}\right)\right]\;p\left(...I_{i},B_{i},U_{i}...\right). \end{array}$$

The expectation value for the total number of ignorant individuals in the population at a given time *t* is defined by

(2) $$\begin{array}{ccc} \langle I\rangle & = & \sum_{I_{1}=0}^{1}\sum_{B_{1}=0}^{1}\sum_{U_{1}=0}^{1}\sum_{I_{2}=0}^{1}...\sum_{U_{N}=0}^{1}\;\left(\sum_{i=1}^{N}\;I_{i}\right)\cdot p\left(I_{1},B_{1},U_{1},I_{2},...,U_{N},t\right), \end{array}$$

and its time evolution is given by

(3) $$\begin{array}{ccc} \frac{d}{dt}\langle I\rangle & = & \sum_{I_{1}=0}^{1}\sum_{B_{1}=0}^{1}\sum_{U_{1}=0}^{1}\sum_{I_{2}=0}^{1}...\sum_{U_{N}=0}^{1}\;\left(\sum_{i=1}^{N}\;I_{i}\right)\cdot \frac{d}{dt}p\left(I_{1},B_{1},U_{1},I_{2},...,U_{N},t\right). \end{array}$$

Inserting the master equation into Equation (3), after some computations, we obtain the dynamic equation for the mean quantity of ignorant individuals in the population:

(4) $$\begin{array}{ccc} \frac{d}{dt}\langle I\rangle & = & -\beta \;{\langle IB\rangle }_{1}-\nu \langle I\rangle -\mu \langle I\rangle +\mu \left(\langle I\rangle +\langle B\rangle +\langle U\rangle \right). \end{array}$$

Similarly, for the expectation value of the total number of believers and unbelievers, we obtain the following dynamic equations:

(5) $$\begin{array}{ccc} \frac{d}{dt}\langle B\rangle & = & \beta \;{\langle IB\rangle }_{1}-\gamma \langle B\rangle -\mu \langle B\rangle \end{array}$$

(6) $$\begin{array}{ccc} \frac{d}{dt}\langle U\rangle & = & \gamma \langle B\rangle +\nu \langle I\rangle -\mu \langle U\rangle . \end{array}$$

The dynamics of the first moments depend on the second moment:

$$\begin{array}{ccc} {\langle IB\rangle }_{1} & = & \sum_{I_{1}=0}^{1}\sum_{B_{1}=0}^{1}\sum_{U_{1}=0}^{1}\sum_{I_{2}=0}^{1}...\sum_{U_{N}=0}^{1}\;\left(\sum_{i=1}^{N}\sum_{j=1}^{N}\;{\left(J^{1}\right)}_{ij}I_{i}B_{j}\right)\cdot p\left(I_{1},B_{1},U_{1},I_{2},...,U_{N},t\right) \end{array}$$

which is the mean number of ignorant and believer neighbors. We can now proceed by computing the dynamic equation for the second moment ${\langle IB\rangle }_{1}$, or we can close the ODE system (4)–(6) by approximating ${\langle IB\rangle }_{1}$ by a mathematical formula involving only the first moments 〈I〉, 〈B〉, and 〈U〉. Here, we close the ODE system (4)–(6) using the mean-field approximation.

Let us assume that the individuals in the population are distributed in a regular network, where all individuals have the same number of neighbors *Q*, and hence,

$$\sum_{j=1}^{N}J_{ij}=Q\;.$$

In the mean-field approximation, the exact number of believers who are neighbors of a certain individual *i* is approximated by the average of the number of believers in the entire population:

$$\sum_{j=1}^{N}J_{ij}B_{j}\approx Q\frac{\langle B\rangle }{N},\;\forall \;i=1,...,N.$$

Hence, the second moment ${\langle IB\rangle }_{1}$ is approximated by

$${\langle IB\rangle }_{1}\approx \frac{Q}{N}\langle I\rangle \langle B\rangle ,$$

and the ODE system (4)–(6) transforms into the closed system

(7) $$\begin{array}{ccc} \frac{d}{dt}\langle I\rangle & = & -\beta \;\frac{Q}{N}\langle I\rangle \langle B\rangle -\nu \langle I\rangle -\mu \langle I\rangle +\mu \left(\langle I\rangle +\langle B\rangle +\langle U\rangle \right) \end{array}$$

(8) $$\begin{array}{ccc} \frac{d}{dt}\langle B\rangle & = & \beta \;\frac{Q}{N}\langle I\rangle \langle B\rangle -\gamma \langle B\rangle -\mu \langle B\rangle \end{array}$$

(9) $$\begin{array}{ccc} \frac{d}{dt}\langle U\rangle & = & \gamma \langle B\rangle +\nu \langle I\rangle -\mu \langle U\rangle . \end{array}$$

We observe that more complex ODEs can be obtained by using higher-order moment closures (see [18,20]).

Next, let the normalized state variables I(t)=〈I〉/N, B(t)=〈B〉/N and U(t)=〈U〉/N denote the mean densities of ignorant, believer, and unbeliever individuals in the population; then, we normalize the time scale τ=(γ+μ)t by the mean believing/spreading period 1/(γ+μ). Hence, I(τ)+B(τ)+U(τ)=1 and Equations (7)–(9) are rescaled to the following ODE system:

(10) $$\begin{array}{ccc} \frac{dI}{d\tau } & = & -R_{0}BI-\left(s+f\right)I+f \end{array}$$

(11) $$\begin{array}{ccc} \frac{dB}{d\tau } & = & R_{0}BI-B \end{array}$$

(12) $$\begin{array}{ccc} \frac{dU}{d\tau } & = & (1-f)B+sI-fU\;; \end{array}$$

where

(a) f=μ/(γ+μ)>0, typically very small, is the mean birth and death rates in the time unit given by the mean believing/spreading period (τ);
(b) s=ν/(γ+μ) is the information search rate in the time unit (τ); and
(c) $R_{0}=\beta Q/\left(\gamma +\mu \right)$ is the so-called basic reproductive number $R_{0}$ (see [21]) in epidemiological models: i.e., $R_{0}$ is the rate at which the expected number of ignorant individuals become believers through the influence of the expected number of believer/spreader individuals in the time unit (τ).

### Stable Stationary States

Let the stationary values of ignorants, believers, and unbelievers of the rumor be denoted by $I^{*}$, $B^{*}$, and $U^{*}$, respectively.

The stationary states of the ODE system (10)–(12) are given by

(13) $$\begin{array}{c} I_{0}^{*}=\frac{f}{s+f},\;B_{0}^{*}=0\;and\;U_{0}^{*}=\frac{s}{s+f}, \end{array}$$

and by

(14) $$\begin{array}{ccc} I^{*} & = & \frac{1}{R_{0}} \end{array}$$

(15) $$\begin{array}{ccc} B^{*} & = & f\left(1-\frac{1}{R_{0}}\right)-\frac{s}{R_{0}}\geq 0 \end{array}$$

(16) $$\begin{array}{ccc} U^{*} & = & \left(1-f\right)\left(1-\frac{1}{R_{0}}\right)+\frac{s}{R_{0}}. \end{array}$$

**Figure 2:**
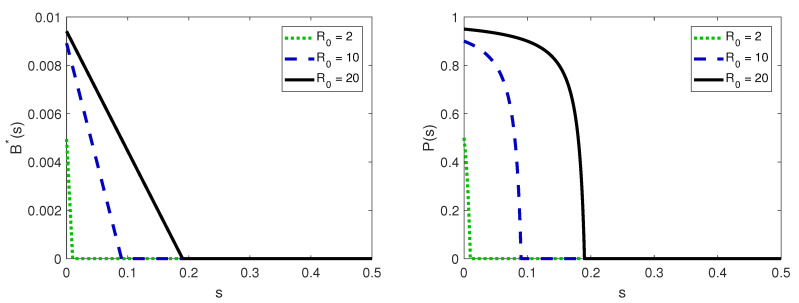
(**left**) The stationary value of believers $B^{*}\left(s\right)$ and (**right**) the probability that an ignorant individual does not search for real information to become believer P(s), which depends on the information search rate *s*. The other parameter is f=0.01.

From Equation (15), we observe that the believers’ stationary state decreases linearly with the information search rate *s* (see also Figure 2), and the *critical information search rate*, which is the rate at which the believers’ stationary state vanishes, is

(17) $$\begin{array}{ccc} s_{C} & = & f(R_{0}-1). \end{array}$$

Since *f* is a small number, we assume in this paper that $0<s_{C}=f\left(R_{0}-1\right)<1$. We observe that the stationary states $\left(I^{*},B^{*},U^{*}\right)$ only hold for $s\leq s_{C}$ because of the natural restriction that $B^{*}\geq 0$. If $s=s_{C}$, then there is a single equilibrium $\left(I_{0}^{*},B_{0}^{*},U_{0}^{*}\right)=\left(I^{*},B^{*},U^{*}\right)$.

**Lemma** **1.** For $s<s_{C}$, the stationary states $\left(I_{0}^{*},B_{0}^{*},U_{0}^{*}\right)$ are unstable, and the stationary states $\left(I^{*},B^{*},U^{*}\right)$ are stable. Furthermore, for $s>s_{C}$, the stationary states $\left(I_{0}^{*},B_{0}^{*},U_{0}^{*}\right)$ are stable.

**Proof.**  The Jacobean matrix of the ODE system (10)–(12) is given by

$$J\left(I,B,U\right)=\left[\begin{array}{ccc} -R_{0}\;B-s-f & -R_{0}\;I & 0\\ R_{0}\;B & R_{0}\;I-1 & 0\\ s & 1-f & -f \end{array}\right]$$

The eigenvalues of the Jacobean matrix $J(I_{0}^{*},B_{0}^{*},U_{0}^{*})$ are

$$\lambda_{1}=-f\;,\;\lambda_{2}=-s-f\;\mathrm{and}\;\lambda_{3}=\frac{f(R_{0}-1)-s}{s+f}\;.$$

Hence, all eigenvalues have a negative real part if and only if $s>f\left(R_{0}-1\right)=s_{C}$. The eigenvalues of the Jacobean matrix $J(I^{*},B^{*},U^{*})$ are

$$\begin{array}{ccc} & & \lambda_{1}=-f\;,\;\lambda_{2}=-1/2\;fR_{0}-1/2\;\sqrt{f^{2}{R_{0}}^{2}+4\;s+4\;f-4\;fR_{0}}\;\mathrm{and}\;\\ & & \lambda_{3}=-1/2\;fR_{0}+1/2\;\sqrt{f^{2}{R_{0}}^{2}+4\;s+4\;f-4\;fR_{0}}\;. \end{array}$$

Hence, all eigenvalues have a negative real part if and only if

$$f^{2}R_{0}^{2}>f^{2}{R_{0}}^{2}+4\;s+4\;f-4\;fR_{0}.$$

This is equivalent to $s<f\left(R_{0}-1\right)=s_{C}$.  □

## 3. Nash and Evolutionarily Stable Information Search Strategies

In this section, we consider a game in which individuals have to decide between searching and not searching for real information to avoid believing the false rumor. Here, we define the Nash and evolutionarily stable information search strategies (see [7,10,11]).

*S* denotes the probability that an ignorant individual will choose to search for information. This probability *S* is the individual’s information search strategy in the game. The uptake level of searching for information in the population is the proportion of individuals who will choose to search for real information, i.e., the mean of all information search strategies. We denote the uptake level of searching for information by *s*, i.e., the population information search strategy.

Let $b_{L}$ and $c_{L}$ denote the benefits and the costs of searching for information, respectively, and let $v=b_{L}-c_{L}$ denote the value of the information search. We define the payoff of an ignorant individual who searches for real information and does not believe in the false rumor by *v*.

Let $b_{B}$ and $c_{B}$ denote the benefits and the costs of believing the rumor, respectively, and let $y=b_{B}-c_{B}$ denote the impact measure that assesses the risk of believing the rumor.

Let P(s) denote the probability that an ignorant individual, who does not search for real information, becomes a believer for a proportion *s* of individuals in the population who search for information. The probability P(s) uses the stable stationary states of ignorant and believer individuals computed in Lemma 1:

(18) $$\begin{array}{ccc} P(s) & = & \frac{R_{0}B^{*}I^{*}}{R_{0}B^{*}I^{*}+fI^{*}}=\frac{f(R_{0}-1)-s}{fR_{0}-s}\;,\;if\;s<s_{C}. \end{array}$$

If $s\geq s_{C}$, then $B^{*}=0$, and thus, P(s)=0 (see Figure 2). In particular, $P\left(0\right)=\left(R_{0}-1\right)/R_{0}$. We define the payoff of an ignorant individual who does not search for real information and believes the rumor by

yP(s).

The *expected information search payoff* E(S,s) of an individual with an information search strategy *S* in a population with an information search strategy *s* is

(19) $$\begin{array}{ccc} E(S,s) & = & vS+yP(s)(1-S)\\ & = & yP(s)+S(v-yP(s)). \end{array}$$

Nash and evolutionarily stable information search strategies are the typical strategies studied in game theory (see [10,12]).

A population information search strategy $s=S^{*}$ is an *information search Nash equilibrium* if

(20) $$\begin{array}{c} \Delta_{S^{*}\to S^{\prime }}=E\left(S^{\prime },S^{*}\right)-E\left(S^{*},S^{*}\right)\leq 0\;, \end{array}$$

for every strategy $S^{\prime }\in \left[0,1\right]$. By Equation (19), an information search strategy $S^{*}$ is a Nash equilibrium if and only if

$$\left(S^{\prime }-S^{*}\right)\left(v-yP\left(S^{*}\right)\right)\leq 0.$$

Let $W\equiv y\left(R_{0}-1\right)/R_{0}$ be the threshold for believing a rumor, where $\left(R_{0}-1\right)/R_{0}=P\left(0\right)$. The remark below follows, for instance, from Lemma 1 in [10].

**Remark** **1.** An information strategy $S^{*}$ is a Nash equilibrium if and only if $S^{*}$ satisfies one of the following conditions:

*(a)*  $S^{*}=0$ and v≤W, with
  E(0,0)=W;or
*(b)*  $S^{*}\in \left(0,1\right)$ and $v=yP(S^{*})$, with $P\left(S^{*}\right)<P\left(0\right)$ and
  $$E\left(S^{*},S^{*}\right)=yP\left(S^{*}\right)=v;\;\;\;\;\mathrm{or}$$
*(c)*  $S^{*}=1$ and v≥0, with
  E(1,1)=v.

**Figure 3:**
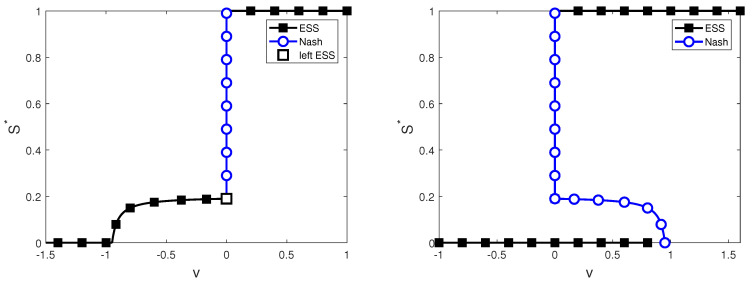
Nash and evolutionarily stable information search strategies *s*, depending on the information search values *v*. On the (**left**), a negative impact measure y=−1 is considered, and on the (**right**), a positive impact measure y=1 is considered. The blue line corresponds to Nash equilibria (that are not ESI strategies), and the black line corresponds to evolutionarily stable information (ESI) search strategies. Other parameter values: $R_{0}=20$, f=0.01, and $s_{C}=0.19$.

Hence, for every $S^{*}>0$, $E(S^{*},S^{*})=v$ is constant, with |v|<|W|. We observe that (i) for every $S^{*}\in \left(0,s_{C}\right)$, $P(S^{*})>0$, and (ii) for every $S^{*}\in \left[s_{C},1\right)$, $P(S^{*})=0$, and thus, $E(S^{*},S^{*})=0=v$. In Figure 3, we plot the Nash information search strategies $s=S^{*}$ for each mixed Nash strategy with the value of information v=yP(s) and for pure Nash strategies $S^{*}=0$ and $S^{*}=1$.

To define an evolutionarily stable information search strategy, we start by assuming that all individuals in the population opt for an individual information search strategy *S*. If a group of size ε chooses a different individual information search strategy $S^{\prime }$, then the population information search strategy becomes

$$\begin{array}{c} s\left(\varepsilon \right)=\left(1-\varepsilon \right)S+\varepsilon S^{\prime }. \end{array}$$

A population information search strategy $S^{*}$ is a *left evolutionarily stable information search strategy* if there is a $\varepsilon_{0}>0$ such that for every $\varepsilon \in (0,\varepsilon_{0})$ and for every $S^{\prime }<S^{*}$,

$$\begin{array}{c} \Delta E_{S^{*}\to S^{\prime }}\left(s\left(\varepsilon \right)\right)=E\left(S^{\prime },s\left(\varepsilon \right)\right)-E\left(S^{*},s\left(\varepsilon \right)\right)=\left(S^{\prime }-S^{*}\right)\left(v-yP(s(\varepsilon ))\right)<0\;. \end{array}$$

The definition of a *right evolutionarily stable information search strategy* is similar. A population information search strategy $S^{*}$ is an *evolutionarily stable information search strategy* if it is a left and right evolutionarily stable strategy.

**Theorem** **1.** A Nash search strategy $S^{*}$ is an evolutionarily stable information (ESI) search strategy if and only if $S^{*}$ satisfies one of the following conditions:

*(i)*  *For positive impact measures y≥0,*
  *(a)*  $S^{*}=0$ and v<yP(0); or
  *(b)*  $S^{*}=1$ and v>0.
*(ii)*  *For negative impact measures y≤0,*
  *(a)*  $S^{*}=0$ and v≤yP(0); or
  *(b)*  $S^{*}\in \left(0,s_{C}\right)$ and $v=yP(S^{*})$; or
  *(c)*  $S^{*}=1$ and v>0.
Moreover, $S^{*}$ is a Nash equilibrium and a left (and not a right) evolutionarily stable information search strategy if and only if $S^{*}=s_{C}$, v=0, and y>0.

Hence, $S^{*}$ is a Nash equilibrium and not an evolutionarily stable information search strategy if $S^{*}\in \left[0,1\right]$ and $v=yP(S^{*})$ and y>0.

In Figure 3, we plot the evolutionarily stable information search strategies $s=S^{*}$ for each value *v* of searching for information.

**Proof.**  The proof follows from Lemma 2 in [10], noting that *v* is negative and $P(S^{*})$ is strictly decreasing for $S^{*}\in \left(0,s_{C}\right)$.  □

## 4. Optimal Strategies

In this section, we compute the optimal information (OI) search strategy for every value of searching for information and every value of the rumor impact measure, under the assumption that all individuals adopt the same information search strategy s=S (homogeneous strategy). Let $s_{C}=f\left(R_{0}-1\right)$ be the critical information search strategy. Let

$$\tilde{E}\left(s\right)\equiv \tilde{E}\left(s;v\right)=vs+yP\left(s\right)\left(1-s\right),$$

for $0\leq s\leq s_{C}$.

**Lemma** **2.** *Assume that f is sufficiently small, where $f<1/R_{0}$.*

*(i)*  *${\tilde{E}}^{\prime \prime }\left(s\right)<0$ for positive impact measure values y>0;*
*(ii)*  *${\tilde{E}}^{\prime \prime }\left(s\right)=0$ for null impact measure values y=0; and*
*(iii)*  *${\tilde{E}}^{\prime \prime }\left(s\right)>0$ for negative impact measure values y<0.*

**Proof.**  We have

$$\begin{array}{ccc} {\tilde{E}}^{\prime \prime }\left(s\right) & = & y\left(P^{\prime \prime }\left(s\right)\left(1-s\right)-2P^{\prime }\left(s\right)\right). \end{array}$$

We observe that ${\tilde{E}}^{\prime \prime }\left(s\right)/y<0$ is equivalent to $2P^{\prime }\left(s\right)>P^{\prime \prime }\left(s\right)\left(1-s\right)$. By Equation (18), we have $P^{\prime }\left(s\right)=-f/{\left(fR_{0}-s\right)}^{2}$ and $P^{\prime \prime }\left(s\right)=-2f/{\left(fR_{0}-s\right)}^{3}$. Thus, $2P^{\prime }\left(s\right)>P^{\prime \prime }\left(s\right)\left(1-s\right)$ is equivalent to

$$-2f/{\left(fR_{0}-s\right)}^{2}>-2f/{\left(fR_{0}-s\right)}^{3}.$$

Hence, we conclude that ${\tilde{E}}^{\prime \prime }\left(s\right)/y<0$ is equivalent to $fR_{0}<1$.  □

By Equation (19), the expected information search payoff is given by

(21) $$\begin{array}{c} E\left(s;v\right)\equiv E\left(s,s;v\right)=\left\{\begin{array}{ccc} \tilde{E}\left(s;v\right) & \;if\; & s\leq s_{C}\\ vs & if & s>s_{C} \end{array}\right.. \end{array}$$

**Figure 4:**
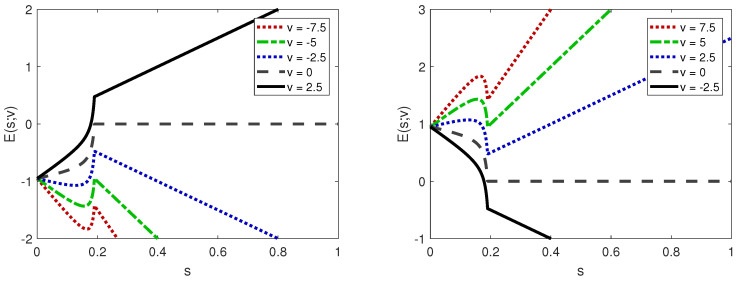
The expected information search payoff, depending on the information search strategy *s*, for different information search values *v*. On the (**left**), a negative impact measure y=−1 is considered, and on the (**right**), a positive impact measure y=1 is considered. Other parameter values: f=0.01, $R_{0}=20$, and $s_{C}=0.19$.

Since $P(s_{C})=0$, we note that $\tilde{E}\left(s_{C};v\right)=v\;s_{C}$, and thus, *E* is a continuous function (see also Figure 4). The optimal information (OI) search strategy (or strategies, eventually) is

$$s_{O}\equiv s_{O}\left(v\right)=\arg\max_{0\leq s\leq 1}E(s;v).$$

The expected payoff of the optimal information search strategy is $E_{O}\left(v\right)=E\left(s_{O}\left(v\right);v\right)$. Let $s_{ESI}\left(v\right)$ denote the evolutionarily stable information search strategy (or strategies, eventually). The expected payoff of the evolutionarily stable information search strategy is $E_{ESI}\left(v\right)=E\left(s_{ESI}\left(v\right);v\right)$. Let $s_{Nash}\left(v\right)$ denote the Nash search strategy (or strategies, eventually) that are not evolutionarily stable information search strategies. The Nash expected payoff is $E_{Nash}\left(v\right)=E\left(s_{Nash}\left(v\right);v\right)$.

### 4.1. The OI Search Strategy for a Positive Impact Measure

Throughout this section, let us assume that the impact measure is positive y>0. Hence, by Lemma 2 (see also Figure 4), for v≠0, $\tilde{E}$ is strictly concave, and thus, the optimal information search strategy is a pure strategy (0 or 1) or a mixed strategy $s_{C}$ or $s_{M}\left(v\right)$, where $s_{M}\left(v\right)$ is the interior maximum point of $\tilde{E}\left(s;v\right)$ (when it exists).

Let $U=y\left(fR_{0}\left(R_{0}-1\right)+1\right)/\left(fR_{0}^{2}\right)$ be the positive information search threshold. Note that 0<W<U.

**Lemma** **3.** *Assume that f is small, where $f<1/R_{0}$. For a positive impact measure y>0, the optimal information (OI) search strategy is*

*(a)*  *for v<W, $s_{O}\left(v\right)=0$, with $E(s_{O}\left(v\right))=W$;*
*(b)*  *$s_{O}\left(W\right)\in \left\{0,1\right\}$, with $E(s_{O}\left(W\right))=W$; and*
*(c)*  *for v>W, $s_{O}\left(v\right)=1$, with $E(s_{O}\left(W\right))=v$.*

For a null impact measure y=0, optimal information (OI) search strategies are similar to those described above, observing that $s_{O}\left(0\right)\in \left[0,1\right]$ (note that W=0).

**Proof.**  Since $\tilde{E}$ is strictly concave, if $E^{\prime }\left(0\right)\leq 0$, then 0 or 1 is the maximum of *E*. Hence, let us compute the following for $E^{\prime }\left(0\right)\leq 0$. The first derivative of the expected payoff is

$$E^{\prime }\left(s\right)=v-y\left(P\left(s\right)+\left(s-1\right)P^{\prime }\left(s\right)\right).$$

Since $P\left(0\right)=\left(R_{0}-1\right)/R_{0}$ and $P^{\prime }\left(0\right)=-1/\left(fR_{0}^{2}\right)$, we have $E^{\prime }\left(0\right)=v-U.$ Hence, $E^{\prime }\left(0\right)\leq 0$ if and only if v≤U. Therefore, for v≤U, 0 is the maximum point of *E* when E(0)≥E(1), and 1 is the maximum point of *E* when E(0)≤E(1). Recall that E(0)=W and E(1)=v. Hence, E(0)≥E(1) if and only if v≤W.

Finally, for v>U>0, let us prove that 1 is the maximum of *E*. This follows from the confirmation that E(s)<E(1) for every $s\leq s_{C}$. We observe that E(s)<E(1) if and only if

$$s<fR_{0}+\frac{yf}{v-y}.$$

Since $s_{C}=f\left(R_{0}-1\right)$, we confirm that the equivalence between

$$s_{C}=f\left(R_{0}-1\right)<fR_{0}+\frac{yf}{v-y}$$

and −y(1−f)<fv holds because of −y(1−f)<0<fv. Hence,

$$s\leq s_{C}<fR_{0}+\frac{yf}{v-y},$$

which concludes the proof.  □

**Remark** **2.** *Assume that f is small, where $f<1/R_{0}$, and the impact measure is positive y>0.*

*(a)*  *$s_{ESI}\left(v\right)=s_{O}\left(v\right)=0$, for v<0;*
*(b)*  *$0=s_{O}\left(0\right)<s_{Nash}\left(0\right)\in \left[s_{C},1\right]$;*
*(c)*  *$0=s_{O}\left(v\right)<s_{Nash}\left(v\right)<1$ and $s_{ESI}\left(v\right)\in \left\{0,1\right\}$, for 0<v<W;*
*(d)*  *$0=s_{Nash}\left(W\right)<s_{ESI}\left(W\right)=1$ and $s_{O}\left(W\right)\in \left\{0,1\right\}$; and*
*(e)*  *$s_{ESI}\left(v\right)=s_{O}\left(v\right)=1$, for v>W.*

For the null impact measure y=0, the comparison is similar to that described above, observing that $s_{O}\left(0\right),s_{Nash}\left(0\right)\in \left[0,1\right]$ (note that W=0).

**Figure 5:**
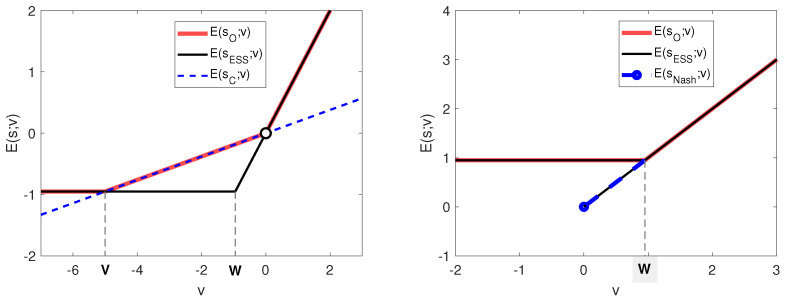
The expected information search payoff E(s;v), depending on the value of the information search *v* for different search strategies *s*: critical search strategy $s_{C}$, OI search strategy $s_{O}$, ESI search strategy $s_{ESI}$, and Nash strategy $s_{Nash}$. On the (**left**), a negative impact measure y=−1 is considered, and on the (**right**), a positive impact measure y=1 is considered. The other parameter values are f=0.01 and $R_{0}=20$. Hence, V=−5 and W=−0.95 (*left*) or W=0.95 (*right*).

By Lemma 3 and Remark 2, (i) for small values of the information search v≤W, the optimal strategy $s_{O}=0$ coincides with the evolutionarily stable information search strategy $s_{ESI}=0$, in which individuals never search for truthful information; (ii) for positive values of the information search v≥W, the optimal strategy $s_{O}=1$ coincides with the evolutionarily stable information search strategy $s_{ESI}=1$, in which individuals always search for truthful information. In Figure 5, we compare the expected payoff of the evolutionarily stable information search $E_{ESI}\left(v\right)$ with that of the optimal information search, denoted by $E_{O}\left(v\right)$.

### 4.2. The OI Search Strategy for a Negative Impact Measure

Throughout this section, let us assume that the impact measure is negative y<0. Hence, by Lemma 2 (see also Figure 4), $\tilde{E}$ is strictly convex, and thus, the optimal information search strategy is a pure strategy (0 or 1) or a mixed strategy $s_{C}$ for v≠0. Let $V=y/(fR_{0})<0$ be the negative information search threshold.

**Lemma** **4.** *Assume that f is small, where $f<1/R_{0}$. For a negative impact measure y<0, the optimal information (OI) search strategy is*

*(a)*  *for v<V, $s_{O}\left(v\right)=0$, with $E(s_{O}\left(v\right))=W$;*
*(b)*  *$s_{O}\left(V\right)\in \left\{0,s_{C}\right\}$, with $E(s_{O}\left(V\right))=W$;*
*(c)*  *for V<v<0, $s_{O}\left(v\right)=s_{C}$, with*
  $$E\left(s_{O}\left(v\right)\right)=vs_{C}=vf\left(R_{0}-1\right)=vW/V;$$
*(d)*  *$s_{O}\left(0\right)\in \left[s_{C},1\right]$, with $E(s_{O}\left(0\right))=0$; and*
*(e)*  *for v>0, $s_{O}\left(v\right)=1$, with $E(s_{O}\left(v\right))=v$;*

**Proof.**  Since $\tilde{E}\left(s;v\right)$ is a linear function in *v*, there is only one value $V=y/(fR_{0})<0$ such that $\tilde{E}\left(0;V\right)=\tilde{E}\left(s_{C};V\right)$. Furthermore, $E\left(0;v\right)>E\left(s_{C};v\right)$ if and only if v<V. (a) If v<V<0, $E\left(0;v\right)>E\left(s_{C};v\right)$ and, by linearity, $E\left(s_{C};v\right)>E\left(1;v\right)$. Hence, $s_{O}\left(v\right)=0$. (b) If v=V<0, $E\left(0;V\right)=E\left(s_{C};V\right)$ and, by linearity, $E\left(s_{C};V\right)>E\left(1;V\right)$. Hence, $s_{O}\left(V\right)=0$ or $s_{O}\left(V\right)=s_{C}$. (c) If V<v<0, $E\left(s_{C};v\right)>E\left(0;v\right)$ and, by linearity, $E\left(s_{C};v\right)>E\left(1;v\right)$. Hence, $s_{O}\left(v\right)=s_{C}$. (d) If v=0, $E\left(s_{C};v\right)>E\left(0;v\right)$ and, by linearity, $E\left(s;0\right)=E\left(s_{C};0\right)$ for all $s\in [s_{C},1]$. Hence, $s_{O}\left(0\right)\in \left[s_{C},1\right]$. (e) If v>0, $E\left(s_{C};v\right)>E\left(0;v\right)$ and, by linearity, $E\left(1;v\right)>E\left(s_{C};v\right)$. Hence, $s_{O}\left(v\right)=1$.  □

Since $V=y/\left(fR_{0}\right)<y\left(R_{0}-1\right)/R_{0}=yP\left(0\right)\equiv W$, we state the following remark.

**Remark** **3.** *Assume that f is small, where $f<1/R_{0}$ and the impact measure is negative y<0.*

*(a)*  *$s_{ESI}\left(v\right)=s_{O}\left(v\right)=0$, for v<V;*
*(b)*  *$s_{ESI}\left(V\right)=0$, $s_{O}\left(V\right)\in \left\{0,s_{C}\right\}$;*
*(c)*  *$s_{ESI}\left(v\right)=0<s_{C}=s_{O}\left(v\right)$, for V<v<W;*
*(d)*  *$0<s_{ESI}\left(v\right)=P^{-1}\left(v\right)<s_{C}=s_{O}\left(v\right)$, for W<v<0;*
*(e)*  *$s_{Nash}\left(0\right),s_{O}\left(0\right)\in \left[s_{C},1\right]$; and*
*(f)*  *$s_{ESI}\left(v\right)=s_{O}\left(v\right)=1$, for v>0;*

By Lemma 4 and Remark 3, (i) for small values of searching for information v≤V, the optimal strategy $s_{O}=0$ coincides with the evolutionarily stable information search strategy $s_{ESI}=0$, in which individuals never search for truthful information; (ii) for positive values of searching for information v>0, the optimal strategy $s_{O}=1$ coincides with the evolutionarily stable information search strategy $s_{ESI}=1$, in which individuals always search for truthful information; (iii) for intermediate values of searching for information V<v<0, the optimal strategy coincides with the critical information search rate $s_{C}$, which eradicates the rumor. This value is above the value given by the evolutionarily stable information search strategy $s_{ESI}$ that is not able to eradicate the rumor and yields a lower expected information search payoff $E_{ESI}\left(v\right)<E_{O}\left(v\right)$ (see Figure 5).

## 5. Evolutionary Information Search Dynamics

Evolutionary information search dynamics is introduced here (see [11,12,13]), under the assumption that all individuals adopt the same information search strategy s=S (homogeneous strategy).

Consider a case in which a small group of individuals of size ε modify their search strategy from the population information search strategy *S* to S+ΔS. The change in the expected information search payoff satisfies

(22) $$\begin{array}{ccc} \frac{\Delta E_{S\to (S+\Delta S)}}{\Delta S} & = & \frac{E(S+\Delta S,s(\varepsilon ))-E(S,s(\varepsilon ))}{\Delta S}=v-yP\left(s\left(\varepsilon \right)\right)\;, \end{array}$$

where s(ε)=(1−ε)S+ε(S+ΔS)=S+εΔS defines the new population search strategy.

Let s(τ) be the population information search strategy adopted at time τ. Hence, we define the *evolutionary information search dynamics* by

(23) $$\begin{array}{ccc} \frac{d\;s}{d\tau } & = & \eta \left(s\right)\lim_{\Delta S\to 0}\frac{\Delta E_{S\to (S+\Delta S)}}{\Delta S}=\eta \left(s\right)\left(v-yP\left(s\right)\right), \end{array}$$

where η(s)≥0 is a smooth map that measures the *information search strategy adaptation speed* of the population.

A point *s* is a *dynamic equilibrium* of the evolutionary information search dynamics if and only if ds/dτ=0. Hence, a point *s* is a dynamic equilibrium if and only if

$$\begin{array}{c} \left(i\right)\;\;\;\;\;\eta (s)=0\;\;\;\;\;\mathrm{or}\;\;\;\;\;\left(\mathrm{ii}\right)\;\;\;\;\;v=yP(s). \end{array}$$

Recall that *f* is assumed to be small, and thus, $s_{C}=f\left(R_{0}-1\right)<1$; P(1)=0, and W=yP(0) is the rumor belief threshold. As usual (see [10]), we assume the following for η: (i) η(s)>0, for all 0<s<1; (ii) if v<W, then η(0)=0 and $\eta^{\prime }\left(0\right)>0$; (iii) if v>W, then η(0)>0; (iv) if v>0, then η(1)=0 and $\eta^{\prime }\left(1\right)<0$; and (v) if v<0, then η(1)>0.

We use the standard definition of left, right, and global attractors for a dynamic equilibrium *p* (see [10]).

**Theorem** **2.** *Assume that f is small, where $f(R_{0}-1)<1$.*

*(i)*  *For negative impact measures y≤0, the dynamic equilibria of the evolutionary information search dynamics are as follows:*
  *(a)*  *for v<0, the evolutionarily stable information search strategy $s_{ESI}\left(v\right)$ is a global attractor;*
  *(b)*  *for v=0, the Nash information search strategies $s_{Nash}\left(v\right)\in \left[s_{C},1\right]$ are equilibria points, and $s_{C}$ is a left (and not right) attractor;*
  *(c)*  *for v>0, the evolutionarily stable information search strategy $s_{ESI}\left(v\right)=1$ is a global attractor.*
*(ii)*  *For positive impact measures y≥0, the dynamic equilibria of the evolutionary information search dynamics are as follows:*
  *(a)*  *for v<W, the evolutionarily stable information search strategy $s_{ESI}\left(v\right)=0$ is an attractor (also global for v<0);*
  *(b)*  *for 0≤v≤W, the Nash information search strategies $s_{Nash}\left(v\right)$ are dynamical equilibria, but not attractors; and*
  *(c)*  *for v>0, the evolutionarily stable information search strategy $s_{ESI}\left(v\right)=1$ is an attractor (also global for v>W).*

**Figure 6:**
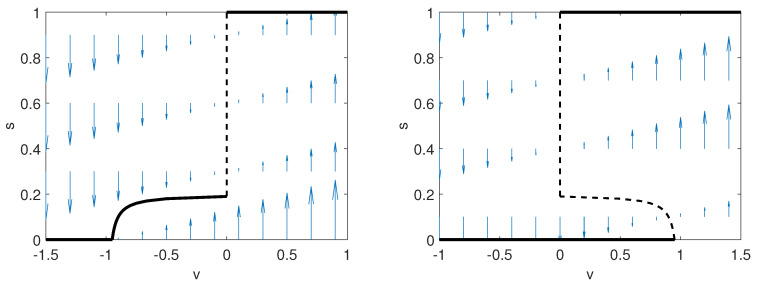
The stable (solid line) and the unstable (dashed line) equilibria of the evolutionary information search dynamics. On the (**left**), a negative impact measure y=−1 is considered, and on the (**right**), a positive impact measure y=1 is considered. Parameter values: f=0.01 and $R_{0}=20$.

In Figure 6, we show the dynamics described above. For advantageous rumors, we observe the existence of a bi-stability region, where the evolutionarily stable information search strategies in which no one searches (persistence of the rumor) or everyone searches (eradication of the rumor) are the attractors, and the Nash equilibria form the boundary of the basins of attraction of the two attractors. Hence, the Nash equilibria are unstable equilibria and are thus not observed (at least for large periods), but have the interesting property of determining the basin of attraction of the attractors. For harmful rumors, we observe that for negative values of the information search v<0, the evolutionary information search dynamic drives the population search strategy to an evolutionarily stable information search strategy that is lower than the critical information search rate $s_{ESI}<s_{C}$. Hence, to eradicate the rumor, a forcing mechanism must be implemented to increase the population search strategy to (or above) the critical information search rate $s_{C}$. For positive values of the information search v>0, the evolutionary information search dynamic drives the population search strategy to the evolutionarily stable information search strategy, in which individuals always search for truthful information $s_{ESI}=1$, and thus, the rumor is eradicated. Hence, a recommendation for legislators and policymakers who aim to eradicate harmful rumors is to make the search for truthful information free or rewarding, i.e., information search value v≥0. Truthful public social media campaigns can help by facilitating access to information.

The proof of the above theorem follows similarly to the proofs of Theorems 6–8 in [10].

**Proof.**  Let y<0 (the proof follows similarly for y>0). To simplify the presentation of the proof, let us introduce the function

F(s)=η(s)(v−yP(s))

such that ds/dτ=F(s).

(a) If v≤yP(0), then η(0)=0, and thus, F(0)=0. Hence, s=0 is an equilibrium point. Since P(s) is decreasing, for every $s^{\prime }\in \left(0,1\right]$, $P\left(s^{\prime }\right)<P\left(0\right)$, and thus, $F(s^{\prime })<0$. Hence,

$$\lim_{\tau \to \infty }s\left(\tau ;s^{\prime }\right)=0\;,$$

and therefore, s=0 is a global attractor.

(b) If yP(0)<v<0 and $s^{*}$ is such that $v=yP(s^{*})$, then $F(s^{*})=0$, and $s^{*}$ is an equilibrium point. Since P(s) is decreasing, for every $s^{\prime }\in \left[0,s^{*}\right)$, $P\left(s^{\prime }\right)>P\left(s^{*}\right)$, and thus, $F(s^{\prime })>0$. Hence,

$$\lim_{\tau \to \infty }s\left(\tau ;s^{\prime }\right)=s^{*}\;,$$

and therefore, $s^{*}$ is a left attractor in $\left[0,s^{*}\right)$. For every $s^{\prime }\in \left(s^{*},1\right]$, $P\left(s^{\prime }\right)<P\left(s^{*}\right)$, and thus, $F(s^{\prime })<0$. Hence,

$$\lim_{\tau \to \infty }s\left(\tau ;s^{\prime }\right)=s^{*}\;,$$

and therefore, $s^{*}$ is a right attractor in $\left(s^{*},1\right]$. Hence, $s^{*}$ is a global attractor.

(c) For every $s^{*}\in \left[s_{C},1\right]$, $P(s^{*})=0$, and thus, $F(s^{*})=0$ if v=0. Hence, $s^{*}\in \left[s_{C},1\right]$ are equilibria points. Since P(s) is decreasing, for every $s^{\prime }\in \left[0,s_{C}\right)$, $P\left(s^{\prime }\right)>P\left(s_{C}\right)=0$, and thus, $F(s^{\prime })>0$. Hence,

$$\lim_{\tau \to \infty }s\left(\tau ;s^{\prime }\right)=s_{C}\;,$$

and therefore, $s_{C}$ is a left attractor in $\left[0,s_{C}\right)$.

(d) If v>0, then η(1)=0, and thus, F(1)=0. Hence, s=1 is an equilibrium point. Since P(s) is decreasing, for every $s^{\prime }\in \left[0,1\right)$, $P\left(s^{\prime }\right)>P\left(1\right)$, and thus, $F(s^{\prime })>0$. Hence,

$$\lim_{\tau \to \infty }s\left(\tau ;s^{\prime }\right)=1\;,$$

and therefore, s=1 is a global attractor.  □

## 6. Conclusions

In this paper, we present a rumor spreading model with potential information searching in a population where individuals can be ignorants, believers, or unbelievers of the rumor. Depending on whether the impact measure, which assesses the risk of believing the rumor (fake news), is positive or negative and on the value of searching for information, we introduce an expected payoff or utility for the individuals. We derive all of the Nash and all of the evolutionarily stable information search strategies. Furthermore, we introduce evolutionary information search dynamics, whose attractors are evolutionarily stable information search strategies.

For advantageous rumors, we observe the existence of a bi-stability region, where the evolutionarily stable information search strategies are either to fully search for truthful information or not search at all. For harmful rumors, we observe that there is a single evolutionarily stable information search strategy by which individuals decide, or not, to search for information. When the benefits of searching for information outweigh the costs, i.e., the value of information search is positive, the evolutionarily stable information search strategy is to search for information with a probability of 1. However, when the value of the information search is negative, the evolutionarily stable information search strategy is smaller than the optimal information search strategy that eradicates the rumor. In this case, unfortunately, the rumor persists. The persistence of false rumors may be quite dangerous and lead to extensive damage to the individual, as well as to all of society. For example, when a disease is spreading, some cases of vaccination with moderate side-effects can be inflated by social media, provoking fear in the population and leading to a large proportion of individuals deciding against vaccination. In an outbreak with a large transmission rate, such as COVID-19, decisions against vaccination are a major contributor to the spread of the disease and so are quite harmful to all. A recommendation for legislators and policymakers who aim to eradicate harmful rumors is to make the search for truthful information free or rewarding.

The population is assumed to be distributed in a regular spatial network, where all individuals have the same number of neighbors, and thus, all of them can equally spread the rumor. This model will be the basis for future works that involve different and more complex spatial networks, heterogeneous strategies, and higher moment closure approximations and encompass the routes of modern social media transmission.
